# An Interactive Virtual Reality Tour for Adolescents Receiving Proton Radiation Therapy: Proof-of-Concept Study

**DOI:** 10.2196/11259

**Published:** 2019-03-05

**Authors:** Jorge Galvez, Melanie Eisenhower, William England, Elicia Wartman, Allan Simpao, Mohamed Rehman, Robert Lustig, Michelle Hribar

**Affiliations:** 1 Section of Biomedical Informatics Department of Anesthesiology & Critical Care Medicine Children's Hospital of Philadelphia Philadelphia, PA United States; 2 Department of Anesthesiology & Critical Care Perelman School of Medicine University of Pennsylvania Philadelphia, PA United States; 3 Department of Child Life, Education and Creative Arts Therapy Children's Hospital of Philadelphia Philadelphia, PA United States; 4 Department of Radiation Oncology Perelman School of Medicine University of Pennsylvania Philadelphia, PA United States; 5 Department of Medical Informatics and Clinical Epidemiology Oregon Health & Science University Portland, OR, OR United States

**Keywords:** child guidance, patient simulation, proton therapy, radiotherapy, virtual reality exposure therapy

## Abstract

**Background:**

Child life therapists provide patient education for children undergoing radiation therapy to assist in coping with and understanding their treatment.

**Objective:**

This proof-of-concept study aimed to determine the feasibility of incorporating a 360-degree video tour via a virtual reality system for children scheduled to receive radiation therapy. The secondary objective was to qualitatively describe each subject’s virtual reality experience.

**Methods:**

Children aged ≥13 years scheduled to receive proton radiation therapy were included in the study. Subjects watched the 360-degree video of the radiation therapy facility in an immersive virtual reality environment with a child life therapist experienced in coaching children receiving radiation therapy and completed a survey after the tour.

**Results:**

Eight subjects consented to participate in the study, and six subjects completed the 360-degree video tour and survey. All the enrolled patients completed the tour successfully. Two subjects did not complete the survey. Two subjects requested to pause the tour to ask questions about the facility. Five subjects said the tour was helpful preparation before undergoing proton radiation therapy. Subjects stated that the tour was helpful because “it showed [them] what’s to come” and was helpful to see “what it’s like to lay in the machine.” One subject said, “it made me feel less nervous.” Six subjects stated that they would like to see this type of tour available for other areas of the hospital, such as diagnostic imaging rooms. None of the subjects experienced nausea or vomiting.

**Conclusions:**

The 360-degree video tour allowed patients to explore the treatment facility in a comfortable environment. Participants felt that the tour was beneficial and would appreciate seeing other parts of the hospital in this manner.

## Introduction

A course of radiation therapy consists of one simulation session followed by 10 to 35 daily radiation sessions. Each treatment session can last between 30 and 90 minutes, depending on the treatment setup and delivery. Radiation therapy protocols are carefully designed to match each patient’s condition.

Patients must be placed in the same position and remain perfectly still to ensure the radiation beam targeting achieves the highest accuracy. Many children require an immobilization device, usually an individually molded plastic shell that fits tightly over the face. For young or anxious children, sedation or general anesthesia is often required, whereas adolescents and older children may undergo radiation therapy without general anesthesia [1-3].

Individuals receiving radiation therapy without general anesthesia often work with child life specialists to learn about radiation therapy and the steps involved as well as to develop coping strategies [4]. One of the challenges patients face is learning about the radiation therapy facility. Visiting the facility may not be feasible due to conflicts with radiation treatment schedules and the patient’s availability. Many patients learn about the radiation therapy facility by watching videos or looking at photographs.

Virtual reality technology is becoming increasingly available to consumers for development and delivery of content. One of the leading developers for virtual reality consumer devices such as the Oculus Rift is Oculus (Irvine, CA). Oculus released the first consumer device in April 2016 [5]. Samsung also has introduced a virtual reality platform for mobile devices such as the Galaxy phones. Virtual reality technology will continue to evolve with new devices over the coming months and years. Virtual reality applications in healthcare are flourishing. Applications have been developed and studied for the evaluation and treatment of anxiety, pain, and other conditions [6-11]. The consumer market for virtual reality technology is becoming increasingly accessible for both consumers and developers. However, virtual reality has its own set of problems. One of the main limitations of virtual reality is that users may experience dizziness, motion sickness, nausea, or vomiting [12]. Furthermore, virtual reality headsets consist of two eyepieces for binocular vision. The specific hardware configuration of a viewer may affect the end-user experience for individuals who cannot see through both eyepieces simultaneously, such as young children.

The primary objective of this study was to determine the feasibility of using a virtual reality headset—the Oculus Rift Development Kit—to deliver a 360-degree video tour of the radiation oncology facility to patients scheduled to receive proton radiation who are eligible for radiation therapy without general anesthesia. The secondary objective was to qualitatively describe the patients’ impression of the 360-degree video tour through a virtual reality system.

## Methods

### Design

This single-site study was conducted in full accordance with all applicable research policies and procedures of the Children’s Hospital of Philadelphia, Hospital of the University of Pennsylvania, and Oregon Health & Science University Institutional Research Boards. The study was approved by the Institutional Review Boards in The Children’s Hospital of Philadelphia and the Hospital of the University of Pennsylvania. All patients and parents provided informed consent and assent to participate in the study.

Study subjects and a parent or guardian provided informed consent and assent before enrollment. This proof-of-concept study evaluated the feasibility of delivering a tour of the Roberts Proton Therapy Center in the Perelman Center for Advanced Medicine (Philadelphia, PA) to pediatric patients with a virtual reality headset.

### Subjects

After the tour, the subjects completed a questionnaire to describe their experience (Table 1). The inclusion criteria for subjects were as follows: proton beam radiation therapy scheduled for the patient, age ≥ 13 years, and English as the primary language. The exclusion criteria were presence of motion sickness, seizure disorder, developmental delay, claustrophobia, cranial incisions with surgical dressings, pain over the scalp or areas that may come in contact with the virtual reality headset, or radiation therapy scheduled to be delivered under general anesthesia. In addition, patients with visual impairment such as diplopia were excluded from enrollment. The virtual reality headset can work with corrective lenses; therefore, 20/20 vision was not a prerequisite. However, children undergoing radiation therapy may have neurologic and ophthalmic conditions that affect vision and may pose a challenge when using a virtual reality system. An interim analysis was performed after three patients completed the protocol, and the protocol was carried out to the conclusion of the study. The trial would have been stopped if two of three subjects experienced side-effects from the virtual reality tour, such as nausea, vomiting, dizziness, or any other symptoms at the time of the interim analysis. The primary endpoint was successful completion of the 360-degree video tour using the virtual reality system and subsequently, the questionnaire.

### The 360-Degree Video Tour and Virtual Reality System

**Figure 1:**
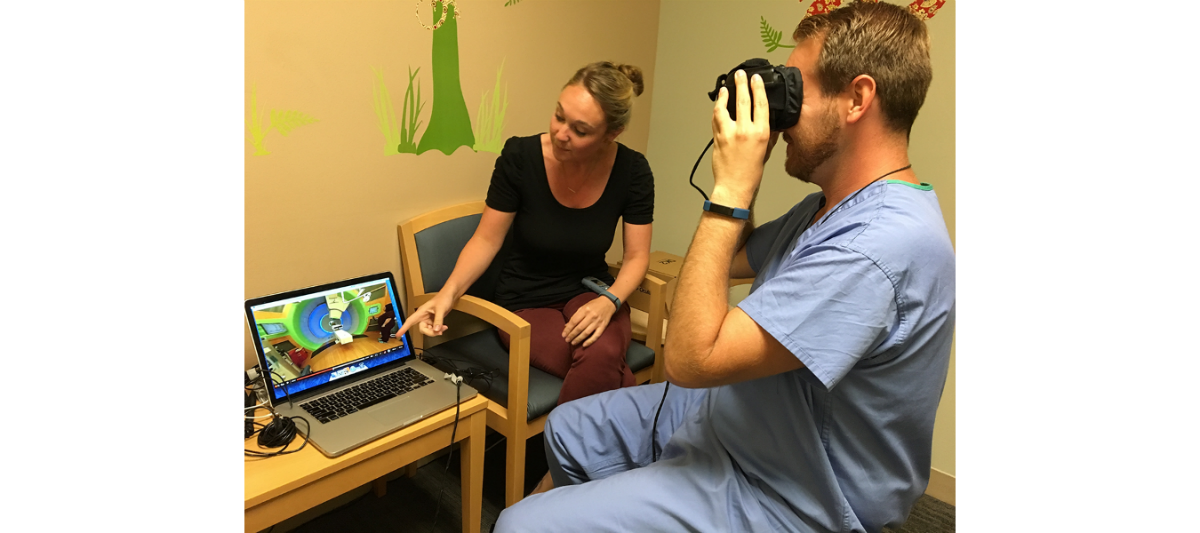
The virtual reality tour is delivered via a laptop and headset in a pediatric consultation room. The child life therapist can control video playback from the laptop. The user can virtually look around the room using the headset. The child life therapist describes each scene as the subject experiences them with the headset. The elastic heads traps on the virtual reality headset were not used. Therefore, the subject had to hold on to the viewer throughout the tour. Subjects were allowed to remove the device if they experienced any discomfort.

A study member demonstrated the use of the equipment and headset to each subject. The subject then held the virtual reality headset over his/her eyes and experienced the 360-degree video tour of the radiation therapy facility in the presence of a child life therapist experienced in pediatric proton therapy (Figure 1). The child life therapist controlled the 360-degree video playback from a laptop (MacBook Pro, Apple Inc, Cupertino, CA) that allowed pausing and replaying of specific portions of the video. The 360-degree video tour footage included the building entrance, elevators, waiting room, changing rooms, corridors, and proton treatment vault, and the tour was approximately 5-7 minutes in duration. Upon completion of the 360-degree video tour, the subject completed a questionnaire to describe his/her experience.

### Equipment

**Figure 2:**
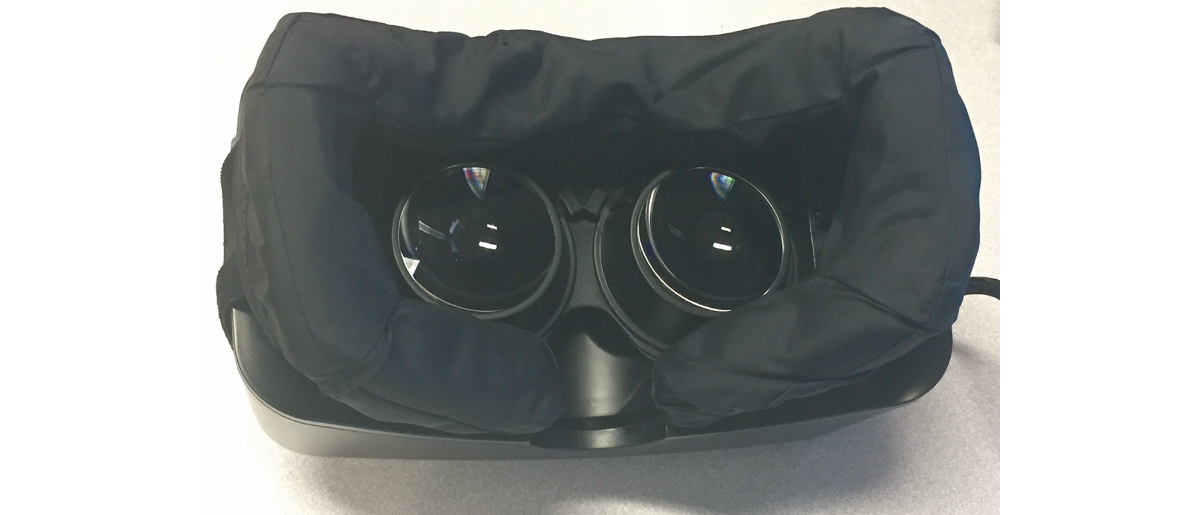
The Oculus Rift DK2 headset with a custom nylon cover over the foam face pads. The device was modified by installing a waterproof cover to facilitate cleaning with alcohol solutions. The elastic straps were secured over the front of the device to allow users to wear the device without using the straps.

The 360-degree video tour was projected using an Oculus Rift Development Kit 2 (Oculus) and a MacBook Pro (Apple Inc). The Oculus Rift viewer has foam face pads that pose an infection risk. Per the hospital’s recommendations for infection control, we fitted a nylon cover on the foam pads (Figure 2). Furthermore, the head straps were secured to prevent contact with the subject’s skin (Figure 2). The nylon cover and Oculus Rift were cleaned between use with hospital-grade disinfectant wipes. The lenses were cleaned with single-use lint-free cloths.

**Table 1:** The posttour questionnaire and answer choices. Subjects were approached to complete this questionnaire immediately after the virtual reality tour.

Question		Answer choices
1	How many times did you pause or stop the video?	0/1/2/3 and why?
2	How many times did you rewind the video?	0/1/2/3 and why?
3	Did you complete the VRT^a^?	Yes/no. If no, why not?
4	Do you think the VRT might help you to prepare to go through proton therapy?	Yes/no/not sure
5	Was there any part of the VRT that was really helpful?	Yes/no. If yes, explain
6	Was there any part of the VRT that was not helpful?	Yes/no. If yes, explain
7	Did you have any other questions about the Proton Therapy Center after watching the VRT?	Yes/no. If yes, what are they?
8	Did you have any discomfort while watching the VRT?	Yes/no
8.1	If yes, what type of discomfort?	N/A^b^
8.2	If yes, did you continue watching?	Yes/no
8.3	What did you do to improve your comfort?	Nothing, adjust headset, pause and resume watching VRT, stopped, or other (______)
9	Would you like to watch a VRT that is similar to this one for other areas or procedures in the hospital?	Yes/might/no/I don't know/other areas or procedures you would suggest (_____)
10	Would you recommend that others watch the VRT?	Yes/no/not sure

^a^VRT: virtual reality tour.

^b^N/A: Not applicable.

The video tour was recorded using GoPro Hero 3 Silver Edition (GoPro, San Mateo, CA) and Kodak SP180 (Kodak, Rochester, NY) with guidance from a child life therapist who conducts the patient tours at the facility. Video editing was performed using Final Cut Pro (Version 10.3.1, Apple Inc). The video was displayed using Kolor Eyes 360 Video Player for Mac, version 1.4.1 (Kolor, Francin, France) [13]. The video player allowed the child life therapist to view the tour on the laptop while the subject was using the Oculus Rift.

## Results

A total of eight subjects consented to participate in the study, and six subjects completed the 360-degree video tour through a virtual reality system (Table 2). Two subjects did not return the survey. Of these, one subject attempted to fill out the survey one day after the tour but had difficulty recalling the details of the tour. All patients who started the tour were able to complete it successfully. Two subjects asked to pause the tour to spend more time exploring individual scenes and ask questions about the facility. One subject paused the tour three times to ask additional questions. Five subjects stated that the 360-degree video tour via a virtual reality system was valuable preparation for proton radiation therapy. Subjects said that the tour was helpful because “it showed [them] what’s to come” and it was useful to see “what it’s like to lay in the machine.” One subject said, “it made me feel less nervous.” When asked if there were parts of the tour that were not helpful, four subjects answered “no.” Two subjects said that the parts of the tour that were not helpful were the rooms that they were already familiar with, such as the changing room.

**Table 2:** Survey results of the questionnaire. Eight subjects completed the 360-degree video tour but only six patients completed the survey. Subjects 3 and 7 did not complete the survey. Subject 7 was approached one day after completing the 360-degree video tour and could not recall details of the tour. All subjects who completed the questionnaire said that they did not have additional questions about the facility after watching the 360-degree video in a virtual reality system. Two subjects experienced some discomfort described as “dizziness” or “eye discomfort” while watching the tour; the discomfort was resolved by adjusting the headset, and these patients were able to complete the tour successfully. One subject had trouble holding the headset during the tour. One subject commented that the image projection in the virtual reality headset was not as clear as the image on the computer. Six subjects stated that they would like to see this type of tour for other areas of the hospital, such as magnetic resonance imaging rooms. None of the subjects experienced nausea or vomiting.

Question	Subject 1	Subject 2	Subject 3^a^	Subject 4	Subject 5	Subject 6	Subject 7^a,b^	Subject 8
1	0	0	LTF^c^	0	1 - to explain what was happening	3 - to talk and ask questions	Unevaluable	0
2	0	0	LTF	0	0	0	Unevaluable	0
3	Yes	Yes	LTF	Yes	Yes	Yes	Unevaluable	Yes
4	Yes	Yes	LTF	Yes	Not sure	Yes	Unevaluable	Yes
5	Yes, it was helpful because it showed me what’s to come	Yes, seeing what it’s like to lay in the machine	LTF	Yes, it made me less nervous	Yes, I didn’t know how large it was	No	Unevaluable	Yes, laying down and seeing the gantry move
6	No	Yes, I saw rooms they showed me before in the actual tour	LTF	No	No	Yes, don’t need to see the changing room	Unevaluable	No
7	No	No	LTF	No	No	No	Unevaluable	No
8	No	Yes	LTF	No	No	Yes	Unevaluable	No
8.1	The VRT^d^ was not as clear as the computer	It made my eye feel some discomfort	LTF	—^e^	—	Some dizziness	Unevaluable	—
8.2	Yes	Yes	LTF	—	—	Yes	Unevaluable	—
8.3	Nothing	Adjust headset	LTF			Nothing	Unevaluable	Holding the headset was troublesome
9	Might	Yes	LTF	Might	Yes	Might, for younger patients	Unevaluable	Yes, especially for MRI^f^
10	Yes	Yes	LTF	Yes	Yes	Yes	Unevaluable	Yes

^a^These subjects did not complete the survey.

^b^This subject could not recall details of the tour 1 day after the tour.

^c^LTF: lost to follow-up.

^d^VRT: virtual reality tour.

^e^Not available.

^f^MRI: magnetic resonance imaging.

## Discussion

We demonstrated that incorporation of a 360-degree video tour via a virtual reality system into patient orientation to a pediatric proton therapy facility is feasible. The patients who experienced the tour described the experience positively. All patients enrolled were able to complete the 360-degree video tour and used the virtual reality system. The 360-degree video tour and virtual reality system allowed the patients to experience what it is like to lay down in the proton therapy gantry and watch the machine move from a first person’s perspective. One patient expressed that “[the 360 degree video tour] made me feel less nervous.” Patients, particularly children and adolescents, undergoing radiation therapy may experience anxiety, especially before the treatment begins.

Virtual reality experiences offer an opportunity to improve the patient’s experience. Child life therapists play an integral role in guiding patients through this process, which ultimately spares the patients from receiving general anesthesia for each radiation therapy session. One advantage of the configuration described is that the video footage is viewed simultaneously by the patient and the child life therapist. In this context, the child life therapist could coach the patient through the experience and answer questions as they come up. The child life therapist could also see what the child is looking at and answer questions accordingly as well as guide the child’s attention to details that may have been missed otherwise. Furthermore, the child life therapist had the ability to pause, rewind, and resume the video to allow more time for discussion. In this study, we successfully introduced a 360-degree video tour delivered through a virtual reality system to the child life therapist’s education tools in this field and are exploring other applications throughout the hospital.

The 360-degree video tour and virtual reality system provide a unique first-person perspective of the radiation therapy room in a neutral environment. In addition, the tour allowed patients to experience laying down on the proton therapy machine and watch the machine operate. Five patients highlighted this as the most valuable part of the tour. The 360-degree video tour also offers access to the proton therapy room without interrupting the scheduled treatment sessions. The proton therapy rooms are always in high clinical demand, and patients have limited opportunities to visit the room before their treatment starts. We are exploring options to expand virtual reality tour access to adult patients in the proton therapy center as well as other parts of the hospital. The patients in our study cohort commented that they would also like to see other parts of the hospital in virtual reality. There are endless possibilities for patient-centered virtual reality-immersing experiences aimed at education, anxiolysis, or analgesia.

The 360-degree video tour was designed specifically for the Roberts Proton Therapy Center at the Perelman Center for Advanced Medicine. One of the limitations we encountered was specific to the device used for the tour. The Oculus Rift DK2 projects a low-resolution image, which appears pixelated. However, the video is not limited to virtual reality viewers, as it is compatible with mobile phones, tablets, and computers. The 360-degree video tour is compatible with most commercially available virtual reality viewers. In this study, we limited the virtual reality tour to patients aged ≥13 years according to the device’s manufacturer recommendations. This age limitation is due to the fixed position of the eyepieces and limited range of interpupillary distance for adults. It is possible for younger children to use the device. However, they may not be able to experience the binocular view or may potentially see double images. In fact, a group in Toronto has designed the “Childlife VR” application to introduce the operating room to patients and have successfully applied it with younger patients by using a generic Google cardboard viewer [14,15].

Virtual reality viewers such as the Oculus Rift are designed for personal use; thus, they may not translate well to clinical environments or may not meet hospital infectious disease-control specifications. The Oculus Rift DK2 has a foam pad, which contacts the face around the eyes, and two elastic head straps, which cannot be easily cleaned or disinfected according to our hospital’s specifications. Hospital infectious disease specialists recommended placing a waterproof cover over the foam pad and cleaning it with hospital-grade disinfectant wipes. Generic single-use devices such as disposable cardboard or paper viewers based on the Google Cardboard model can circumvent the cleaning problem [12]. However, viewers made of corrugated cardboard pose an infectious control risk and do not meet our hospital’s requirements.

The device configuration described in this study is time consuming, requires a clean working surface to configure all the necessary equipment, and can take 5 to 15 minutes to set up. Portable virtual reality technology is evolving rapidly, and new devices are constantly entering the market. The virtual reality tour is available as a digital video file and is compatible with any commercially available virtual reality viewer, such as hand-held virtual reality viewers designed for mobile phones or hand-held devices. There are many options available in the market and more will become available. In the meantime, the 360-degree video tour can be delivered via a digital video player such as a computer or tablet in addition to any virtual reality device or 360-degree video player in the market.

Health care providers are constantly evaluating emerging technologies to improve patient care and the patient experience. Virtual reality technology is becoming widely available for consumer use. This technology has great potential for various applications in health care, such as assisting health care providers in introducing patients to the hospital setting and therapy areas such as radiation therapy. Portable virtual reality technology is growing rapidly, and its role in healthcare is evolving quickly. This study provides an example of the feasibility of implementing virtual reality in a pediatric clinical setting to supplement child life efforts and dispel the fear of the unknown. Finally, the 360-degree video tour and virtual reality system provide access to a clinical area that is inaccessible to patients due to the high clinical demand and fast-paced schedules. As virtual reality technology continues to evolve, so will health care providers’ ability to understand its application and improve the patient experience.

## Abbreviations

LTF: lost to follow-up
MRI: magnetic resonance imaging
VRT: virtual reality tour
